# Chronic Peritonitis Following Carditis

**Published:** 1910-01-29

**Authors:** 


### Medicine.

### CHRONIC PERITONITIS FOLLOWING CARDITIS.

A girl aged 12 was admitted into the hospital on July 4. Her chief complaint was distension of the abdomen, which was found to be uniformly enlarged, globular and tense. It was dull on percussion, except at the highest point, and on changing the patient's position this small resonant area shifted always to the highest point. There was no tenderness. On deep palpation it was thought that the liver could be felt, but the spleen could not be made out. The legs were not swollen. There was no jaundice. The bowels acted normally and regularly.

The veins of the face were slightly distended, the eyes were a little prominent, and the lips slightly cyanosed. The external jugular veins were distinctly full, and they pulsated slightly with the systole of the heart, but did not fill from below. The cardiac impulse was obscurely felt in about its natural position; there was no extension of the heart's dulness beyond the normal limits, and no bruit could be heard. The pulse rate was 100, and the pulse itself occasionally intermitted during inspiration, but presented no other abnormality. The respiration rate was slightly quickened, with some dyspnœa on simple exertion. The chest was symmetrical and the epigastric angle was equal upon the two sides, though the angle as a whole was obtuse owing to the abdominal distension. There was impairment of note over the lower two inches of the bases of the lungs, and a few non-consonating rales were heard in the same region. The patient had no cough and no expectoration. The urine was natural, except that upon two occasions it contained a trace of albumen. The head had prominent parietal eminences, but there were no general signs of former rickets. The incisor teeth were natural. There was no pyrexia.

The patient's family history seemed to throw no light upon her condition. From the personal history it appeared that she had enjoyed good health until the age of nine, when she became weak and languid, and was admitted into another hospital for cough. The notes made at that time make it clear that she suffered from acute pericarditis, probably with effusion; no cardiac murmur was heard.

Some months after the pericarditis her abdomen swelled and eventually it was "tapped." Temporary relief followed, but the fluid re-accumulated, and in the two years previous to her final admission she was tapped eight times, and a large amount of fluid was drawn off at each operation.

On July 21 she was tapped and 8½ pints of fluid were removed. This was turbid, highly albuminous, and of specific gravity 1010. The microscope showed that the turbidity was not due to fat, only some amorphous shreds and very few formed elements being present. After the fluid was withdrawn the liver could be felt projecting below the costal margin. It was thought to be enlarged, but it was not tender. Some friction fremitus was perceptible over the liver. The spleen could not be felt. The abdomen gradually refilled. A second tapping was performed on August 21, and the patient was discharged on September 1. From this date until her death 4½ years later she was a regular visitor to the hospital for the purpose of being tapped. It was calculated that in the last seven years of her life this operation was performed 39 times, the fluid withdrawn amounting collectively to some 40 gallons. In its empty condition the abdomen continued without notable change. The bowels acted regularly. The urine contained a cloud of albumen occasionally, but otherwise was normal.

The thoracic organs remained in the condition described until five months before the patient's death, when in July there was cough and dyspnœa and the legs began to get œdematous. There was also some fever, the temperature occasionally rising as high as 103° F. The lung trouble subsided after the abdomen was tapped.

In November the cough was again very troublesome, and the patient reported that during her absence from the hospital she had had "pneumonia" at home. The left side of the chest was now found to be dull on percussion posteriorly, and the finger-tips were decidedly clubbed. From this time the lung trouble increased, as also did the œdema of the legs. Acupuncture of the latter had to be resorted to, but shortly afterwards the patient died of exhaustion and heart failure.

At the post-mortem examination the following condition was found:—

The peritoneum was everywhere smooth, white, sodden-looking, and thickened. In some places it was as much as $\frac{1}{16}$th of an inch thick, dense and opaque. It matted all the abdominal contents together, and yet it could easily be stripped off the liver, spleen, and intestines. The great omentum was almost non-existent. The stomach and intestines showed no internal lesion. The liver had no scarring of its surface. It was clearly not cirrhotic, and the only abnormality noted in it was a moderate degree of nutmeg change. The kidneys were quite natural, and so was the spleen.

The pericardium was adherent both to the heart and to the chest wall, diaphragm and both pleuræ. Both lungs exhibited some compression of their bases; there was a small infarct in the right lung, and there was recent purulent lymph in the lower part of the left pleural cavity. The heart itself was not markedly abnormal in size, and the valves were perfectly sound.

The case illustrates a thing that is not uncommon in children, namely, the occurrence of subacute peritonitis in heart cases. There was no direct proof that the child's pericarditis had been rheumatic, but presumably it was so. It is not very uncommon in children with heart disease, to find ascites without œdema of the legs; it would seem that this ascites is not attributable to "backward pressure," but is rather analogous to the pleurisy and the pericarditis that rheumatic children are still more liable to. The peritoneum having been subacutely inflamed in the first instance, it is not necessary to suppose that it continues to be inflamed all the time the fluid continues to re-accumulate. The original inflammation may subside entirely and yet leave a condition of blockage of all the stomata and lymphatics in the diaphragm, through which the ordinary normal secretion of the peritoneum should escape. When this blockage of the stomata has occurred, repeated re-accumulation of the ascitic fluid is inevitable, even though the actual peritonitis has long since ceased.

Another alternative, that the peritonitis was originally tuberculous, but that the tubercles had resolved long before death, seems unlikely in the face of the fact that tuberculous pericarditis is exceedingly uncommon; and yet in the case in question pericarditis was certainly antecedent to the ascites.

